# Effect of hyperbaric oxygenation on mitochondrial function of neuronal cells in the cortex of neonatal rats after hypoxic-ischemic brain damage

**DOI:** 10.1590/1414-431X20165187

**Published:** 2016-04-26

**Authors:** L. Yang, M.Y. Hei, J.J. Dai, N. Hu, X.Y. Xiang

**Affiliations:** Department of Pediatrics, Third Xiangya Hospital, Central South University, Changsha, Hunan, China

**Keywords:** Hyperbaric oxygenation, Hypoxia-ischemia, Brain, Mitochondrial function, Neonatal, Rat

## Abstract

The timing and mechanisms of protection by hyperbaric oxygenation (HBO) in hypoxic-ischemic brain damage (HIBD) have only been partially elucidated. We monitored the effect of HBO on the mitochondrial function of neuronal cells in the cerebral cortex of neonatal rats after HIBD. Neonatal Sprague-Dawley rats (total of 360 of both genders) were randomly divided into normal control, HIBD, and HIBD+HBO groups. The HBO treatment began immediately after hypoxia-ischemia (HI) and continued once a day for 7 consecutive days. Animals were euthanized 0, 2, 4, 6, and 12 h post-HI to monitor the changes in mitochondrial membrane potential (ΔΨm) occurring soon after a single dose of HBO treatment, as well as 2, 3, 4, 5, 6, and 7 days post-HI to study ΔΨm changes after a series of HBO treatments. Fluctuations in ΔΨm were observed in the ipsilateral cortex in both HIBD and HIBD+HBO groups. Within 2 to 12 h after HI insult, the ΔΨm of the HIBD and HIBD+HBO groups recovered to some extent. A secondary drop in ΔΨm was observed in both groups during the 1-4 days post-HI period, but was more severe in the HIBD+HBO group. There was a secondary recovery of ΔΨm observed in the HIBD+HBO group, but not in the HIBD group, during the 5-7 days period after HI insult. HBO therapy may not lead to improvement of neural cell mitochondrial function in the cerebral cortex in the early stage post-HI, but may improve it in the sub-acute stage post-HI.

## Introduction

Medical research has long focused on understanding the pathophysiology of hypoxic-ischemic brain damage (HIBD) in newborns, which still has a high incidence in the world, causing negative developmental outcomes in children (1). Clinically, though hypothermia has been proved to be effective in reducing hypoxia-ischemia (HI) induced brain damage (2,3), specific treatment to HIBD is still limited, leaving the task of finding new therapeutic methods to be very necessary. The initial insult in HIBD is the deprivation of oxygen (O_2_) to the brain cells. There is a cascade of brain cell damage after HI insult, from O_2_ deprivation, followed by N-methyl-D-aspartate (NMDA) receptor activation and intracellular free calcium accumulation, to mitochondrial dysfunction and other biochemical changes of brain cells, finally resulting in the death of cells via apoptosis or necrosis (4). Any intervention that effectively reverses each step in the cascade may have a potential treatment effect in HIBD. Interventions directed to improving mitochondrial function of injured brain cells has drawn special attention (5). Under any circumstance of hypoxia/ischemia-inducing cell death, mitochondrial dysfunction caused by energy failure is the earliest pathophysiology process (6,7). The usual pathway is: energy failure - free calcium accumulation in the cell - mitochondrial dysfunction - cytochrome C released from the mitochondria - other cascade steps of cell damage - cell necrosis/apoptosis.

Theoretically, hyperbaric oxygenation (HBO) could affect the recovery of mitochondrial function in HIBD, since such a hyperoxic condition will undoubtedly affect cerebral energy metabolism, in which mitochondria plays critical roles mediating cellular responses to HI stress. In the past, HBO has been shown to increase cerebral blood flow and decrease intracranial pressure while increasing oxygen availability to injured brain cells (8). An animal study found that HBO appears to restore mitochondrial function by greatly increasing O_2_ delivery diffusion gradient, which subsequently improves cerebral aerobic metabolism after brain injury (9). We hypothesize that HBO may reduce HI-induced brain injury by affecting the brain cell mitochondrial function. Our aim is also to evaluate the change patterns of mitochondrial function in HBO treatment. In the present study, we used flow cytometer to explore the change of mitochondrial membrane potential (ΔΨm) of neuronal cells in the cortex of neonatal rats after HIBD.

## Material and Methods

The present study was approved by the Medical Ethics Committee of the Third Xiangya Hospital. The study was carried out according to the guidelines for animal experimentation at Central South University, Changsha, Hunan, China.

### Animals and HIBD model

Sprague Dawley rat pups on postnatal day 7 (total of 360, of both genders, mean weight: 12.11±1.19 g) were randomly divided into three groups: normal control group, HIBD group, and HIBD+HBO group. The Rice-Vannucci model of HIBD (10) was established with minor modifications. Briefly, the left common carotid artery was permanently ligated under isoflurane inhalation anesthesia (10). After resting with its dam for 2 h at room temperature, the pup was subjected to humidified 8% O_2_/92% N_2_ hypoxia in an air-tight chamber at 34°C for 2 h (10 pups at a time). The end of HI was considered to be 0 h time-point. This technique has been successfully used in our laboratory for years in a series of HIBD studies (11-13).

### HBO treatment

Series of HBO treatment began after the end of HI insult. Animals (10 pups at a time) were placed in a pressure chamber made of transparent acrylic plastic (inner diameter 25 cm, length 50 cm). The temperature in the chamber was kept at 34°C. HBO-treated animals were pressurized for 15 min to 2-atmosphere absolute pressure (ATA) (0.2 MPa) and maintained for 60 min at O_2_ concentration of no less than 85%. Controls were also transferred into the chamber for the same duration, but not pressurized, and with normal room air. The HBO treatment was given once a day for 7 consecutive days (in 24-h intervals).

### Flow cytometer measurement of ΔΨm

Animals were euthanized by decapitation after giving overdose of phenobarbital, at 0, 2, 4, 6 and 12 h, in order to study the ΔΨm changes at the very early stage after a single dose of HBO treatment, and at 2, 3, 4, 5, 6 and 7 days, in order to study the ΔΨm changes after a series of HBO treatments. Cell suspension of the cerebral cortex was prepared as described previously (14). In detail, cerebral cortex was separated and immersed in Earl's balanced salt iced solution, and then transferred to 2mL of papain digesting solution. The tissues were minced, put on a shaker at 37^o^C and gently shaken. After 30 min, an equal volume of normal physiological medium/bovine serum albumin (NPM/BSA) was added to block the reaction. The mixture was gently triturated with 5 mL pipette over 10 passages. The cell suspension was kept upright on ice for a while and any undigested or aggregated particles were removed after. The solution was centrifuged at a low speed of 300 × *g* at 4^o^C in an Eppendorf centrifuge 5810R (Germany) for 5 min. The supernatant was decanted and the cells were re-suspended in 5 mL of NPM/BSA. After 3 rounds of centrifugation/re-suspension, dissociated cells were finally re-suspended at a density of 2×10^6^ cells/mL in NPM/BSA. The counting for viable cells was done after trypan-blue exclusion.

The ΔΨm was determined by measuring the green fluorescence of Rhodamine123 (Rho123; Sigma, USA). Mitochondria were stained by directly adding a stock solution of Rho123 (10 µM in ethanol) to 1 mL of the cell suspension to a final concentration of 1 µM for 45 min with gentle horizontal agitation in a shaker at 37^o^C under an atmosphere of 95% O_2_/5 % CO_2_. Changes in Rho123 fluorescence were measured by flow cytometer (excitation wavelength at 488 nm and emission wavelength at 525 nm; Cytomics FC500, Beckman Coulter, Inc., USA). As a positive control for ΔΨm depolarization, 1 mL of cell suspension from a randomly chosen animal was preincubated with the uncoupling agent cyanide m-chlorophenylhydrazone (mCICCP, 100 µM, Sigma, USA) before loading Rho123.

### Statistical analysis

Data were analyzed using the SPSS 13.0 software (USA). ANOVA was used to evaluate differences among experimental groups followed by Tukey *post hoc* test. Statistical significance was set at P<0.05.

## Results

### Fluctuating changes of ΔΨm after a single dose of HBO within 24 h

The mean ΔΨm of the normal control group at matched time-points within 24 h was (4.72±0.12) MFL (mean fluorescence level). In the HIBD and HIBD+HBO groups, the ΔΨm of the ipsilateral cortex at each time-point within 24 h was lower than the normal control, and the lowest one was at 0 h in both groups. There was a significant difference between the HIBD and normal control groups, and between the HIBD+HBO and normal control groups. The ΔΨm in both groups had a fluctuating change pattern from 0 to 12 h, showing first a decrease, then a recovery at 2 h, and a decrease again at 12 h. The mean ΔΨm in the HIBD+HBO group was higher than that in the HIBD group at 2, 4, and 6 h, but lower than that in the HIBD group at 0, 12 and 24 h (Table 1). Among these differences, significance was found at 4, 12, and 24 h.

**Table:**
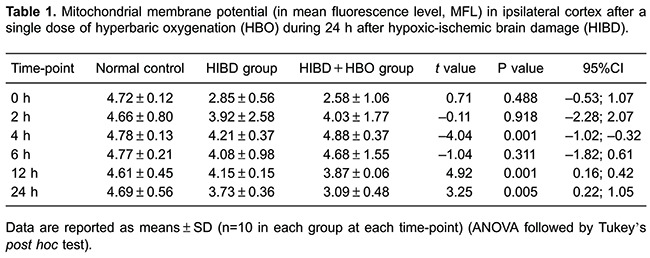

### Recovering ΔΨm changes after daily HBO treatment for 7 days

The mean ΔΨm for the normal control group at matched time-points from 1-7 days after HI was 4.66±0.80 MFL, which was not significantly different from that within 24 h after HI. At 1 to 7 days, the ΔΨm of the ipsilateral cortex of the HIBD group was significantly lower than the normal control group without much difference between each other. The ΔΨm of the HIBD+HBO group at 1, 2, and 3 days was initially lower than of the HIBD group, then it recovered at 4 and 5 days, and it became higher at 6 and 7 days. The difference between the HIBD group and the HIBD+HBO group was significant at 1, 2, 3, 6, and 7 days (P<0.05) (Table 2). The ΔΨm of the HIBD+HBO group at 6 and 7 days was very similar to the normal control (P>0.05).

**Table:**
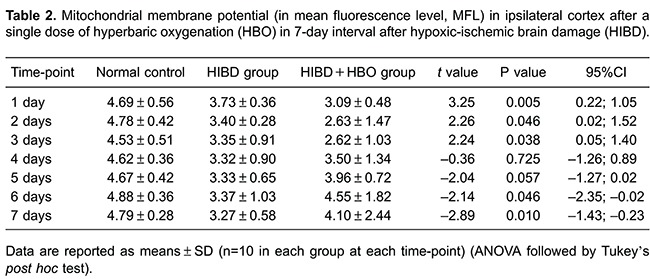

**Figure 1:**
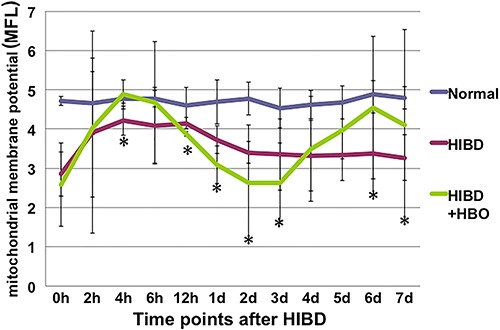
Change in mitochondrial membrane potential (Δψm) in ispilateral cortex of newborn rats after hypoxic-ischemic brain damage (HIBD) and HIBD with daily hyperbaric oxygenation (HBO) treatment for 7 days. The change in ΔΨm (mean fluorescence level, MFL) in both groups showed fluctuating pattern. Within 2 to 12 h after HI insult, ΔΨm of the HIBD group recovered to some extent, but in the HIBD+HBO group it recovered to almost normal levels. A secondary drop (more severe in the HIBD+HBO group) was observed in both groups at 1-4 days after hypoxia-ischemia (HI) insult. A secondary recovery was observed in the HIBD+HBO group only. Data are reported as means ±SD. *P<0.05, significant differences between HIBD and HIBD+HBO groups (ANOVA followed by Tukey’s *post hoc* test).

The change of ΔΨm in the ipsilateral cortex from the normal control, HIBD and HIBD+HBO groups at each time-point is summarized in Figure 1. There was a “drop-recovery-secondary drop-secondary recovery” change pattern after daily HBO treatment for 7 days in the HIBD+HBO group. The secondary recovery was not observed in the HIBD group. The *post hoc* test did not reveal significant differences between time-points.

## Discussion

The main findings of the present study were: ΔΨm of the ipsilateral cortex of the HIBD group within 7 days after HI had a ‘drop-recovery-secondary drop' fluctuation, while in the HBO-treated rat pups the ΔΨm showed a ‘drop-recovery-secondary drop-secondary recovery' change pattern. The secondary drop in the HIBD and HIBD+HBO groups occurred from 12 h to 3 days after HI, and the extent of the secondary drop was more severe in the HIBD+HBO group, indicating that HBO in the early stage after HI might not be a good therapy to improve mitochondrial function in the cerebral cortex. On the other hand, a secondary recovery occurred after 4 days of HBO treatment, elevating ΔΨm of the ipsilateral cortex in the HIBD+HBO group to near normal levels. This might indicate a possible protective effect of HBO treatment in HI-induced brain damage by improving neural cell mitochondrial function. These findings add to the understanding of the effects of HBO in neonatal HIBD, and its relevant mechanisms.

ΔΨm is a commonly used surrogate biomarker for mitochondrial function. After brain injury, there is a cascade of biochemical events leading to mitochondrial dysfunction (15). In the present study, Rho123 was used as a probe for mitochondrial function. Rho123 is a cationic fluorescent dye that binds specifically to the mitochondria of living cells, and it has been used for the estimation of ΔΨm (16). Another commonly used fluorescent probe for estimating ΔΨm is 5,5′,6,6′-tetrachloro-1,1′,3,3′-tetra-ethyl-benzimidazol-carbocyanine iodide (JC-1) (17). Fluorescence is a commonly used bioanalytical method, offering sensitive and quantitative results. The best example of the latter is flow cytometry (18), which was used in the present study. We used the Rice-Vannucci HIBD rat model, which presents the advantage of allowing the ipsilateral hemisphere of the animal to be the internal control (10).

Oxygen delivery depends on a pressure gradient from the alveolar spaces to the blood and finally to the brain tissue itself. Hyperbaric O_2_ increases the vital O_2_ delivery pressure gradient. Brain tissue O_2_ monitoring levels of 200-300 mmHg are recorded with HBO at 1.5 ATA (19), which increases the amount of dissolved O_2_ in the plasma 10-fold normal levels (20). The HBO treatment in the present study was at 2 ATA pressure for 60 min, hence we believe that the amount of dissolved O_2_ in the plasma was higher than 10-fold normal levels. This protocol is one of the most commonly used for standard therapeutic purposes (1.8-2.8 ATA for 60-90 min) (21). The increased amount of oxygen dissolved in the blood results in improvement of a variety of clinical conditions such as hypoxia (22). HBO has been used for multiple neurological diseases (23), including neonates (24). It was reported that a single dose of HBO reduced HI brain injury in neonatal rats (25). The main mechanism of HBO in HIBD might be the favorable influence in the binding of O_2_ in mitochondrial redox enzyme systems, which helps to improve mitochondrial function (26), and to inhibit apoptosis (27).

The findings of the present study are in accordance with other studies. Zhou et al. (9) reported that HBO improved mitochondrial function by preserving ΔΨm and increasing ATP production after fluid-percussion brain injury. Palzur et al. (28) reported that the neuroprotective effect of HBO was mediated by inhibition of the mPTP and subsequent reduction of the mitochondrial pathway of apoptosis. This may explain the mechanism for the recovery of ΔΨm in the ipsilateral cortex in the HIBD+HBO group. In the present study, the more severe drop of ΔΨm at 12 h to 3 days in the HIBD+HBO group indicates a more severe damage in mitochondrial function caused by HBO. This is not in accordance with the findings of a study in transient focal ischemia brain damage in the adult middle cerebral artery rat model, in which HBO was highly efficient in reducing ischemic injury within the first 6 h (29). These results suggest that early application of HBO treatment might not help to reduce brain damage after HI in neonatal rats. We consider that this mechanism is related to tissue reperfusion and free radical damage. After brain injury, reactive oxygen and reactive nitrogen species may be generated by inflammatory cells through several different cellular pathways, including calcium activation of phospholipases, nitric oxide synthase, xanthine oxidase, and the Fenton and Haber-Weiss reactions (30). If cellular defense systems are weakened, increased production of free radicals will lead to oxidation of lipids, proteins, and nucleic acids, which may alter cellular function in a critical way (30). In addition, hyperoxia can also cause potential cerebral toxicity. Brain tissue is especially vulnerable to lipid peroxidation because of its high rate of O_2_ consumption and high content of phospholipids (31). Additionally, the brain has limited natural protection against free radicals - that is, it has limited reactive oxygen species (ROS) scavenging ability, poor catalase activity, and is rich in iron, which is an initiator of ROS generation in brain injury via the Fenton reaction (26).

In the neonatal population, O_2_ toxicity has always drawn neonatologist's attention due to its negative outcomes of retinopathy of prematurity and lung injuries. However, it was reported that HBO at 2.0 ATA for 60 min, including intermittent decompression and compression 2-3 times per 24 h, is a relatively low O_2_ exposure and carries a low risk for O_2_ toxicity in rats (26). In addition, exposure to hyperoxia for 1 h at hyperbaric pressures did not result in the structural changes or abnormal vascularization associated with retinopathy of prematurity in rats (24). Yoles et al. (32) compared the metabolic response, the hemodynamic changes and the electrical activity of dog puppies of different ages during exposure to HBO, and found that younger puppies had higher resistance to HBO-caused brain damage.

The limitations of the present study were: 1) sample size of the groups at each time-point might have not been large enough to reduce the SD of the data. 2) The effect of HBO to HIBD regarding memory or cognitive development was not evaluated. 3) Mechanisms of the ΔΨm fluctuation after HBO treatment, such as NMDA receptor activation or intracellular free calcium accumulation, were not included in the present study. These mechanisms might be our next research considerations.

In conclusion, the timing and mechanisms of HBO protection in HIBD have only been partially elucidated. A fluctuation of mitochondrial function with primary and secondary drops was observed during the 1-4-day period post-HI. There was a secondary recovery of the mitochondrial function after HBO intervention during the 5-7-day period after HI insult. Findings of this study indicate that HBO therapy might not improve neural cell mitochondrial function in the cerebral cortex in the early stages post-HI, but improvement might be achieved in the sub-acute stage post-HI.
